# Nursing Students’ Knowledge and Awareness of Antibiotic Use, Resistance and Stewardship: A Descriptive Cross-Sectional Study

**DOI:** 10.3390/antibiotics8040203

**Published:** 2019-10-30

**Authors:** Andrea Rábano-Blanco, Eva María Domínguez-Martís, Diego Gabriel Mosteiro-Miguéns, Manuel Freire-Garabal, Silvia Novío

**Affiliations:** 1 Galician Public Health Care Service, Milladoiro, Ames, 15895 A Coruña, Spain; andrea4_95@hotmail.com; 2 Galician Public Health Care Service, Health Care Centre of Ordes, Ordes, 15680 A Coruña, Spain; eva.dominguez2@hotmail.com; 3 Badalona Serveis Assistencials, Family and Community Nursing, Health Care Centre of Morera-Pomar, 08915 Badalona, Spain; diegomoste@gmail.com; 4 School of Medicine, University of Santiago de Compostela, 15782 A Coruña, Spain; manuel.freire-garabal@usc.es; 5 Department of Psiquiatry, Radiology, Public Health, Nursing and Medicine, School of Medicine and Dentistry, University of Santiago de Compostela, 15782 A Coruña, Spain

**Keywords:** Anti-bacterial agents, awareness, drug resistance, knowledge, nursing, students

## Abstract

Antibiotic resistance is an emerging worldwide concern with serious repercussions in terms of morbi-mortality. Bearing in mind that the inadequate use of antibiotics, by healthcare staff as well as by the general population, is one of its main causes, a multidisciplinary approach is required to try to combat it. The aim of the present study was to determine nursing students’ knowledge and awareness of antibiotic use, resistance and stewardship. A cross-sectional design was used. A total of 578 nursing students from the University of Santiago de Compostela (Spain), ≥18 years old of both sexes were invited to complete the Spanish version of the questionnaire “Knowledge and awareness of the use, resistance and administration of antibiotics” between February and April 2019. Students had a low level of knowledge about antibiotics, 4.1 (CI95% = 3.4–4.8), especially in relation to antibiotic resistance. As the students were aware of this deficiency, the majority affirmed (>90%) that the current curriculum of nursing degree should have more training on antibiotics and infection control. Nursing staff play an important role in the rational use of antibiotics and as teachers of patients, so their training could be key in fighting antibiotic resistance.

## 1. Introduction

Antibiotics are medicines that fight bacterial infections. They can save lives if they are used correctly, but nowadays antibiotic resistance has become a real problem [1]. Infections by resistant microorganisms do not respond to treatment, so the duration of the disease is prolonged, and the risk of death increases. Moreover, if the treatment fails or if the response to treatment is slow, the patient is contagious for longer, which increases the likelihood of resistant microorganisms being transmitted to other people [2].

The emergence of antibiotic resistance is a natural biological phenomenon, and even though numerous factors have been identified as the cause of this, such as clustering and overcrowding or increased elderly population [2], one of the most important factors is the inadequate use of antibiotics [3], a very worrying situation especially for healthcare professionals with high rate of antibiotic prescription such as primary care dentists [4]. According to the World Health Organization (WHO), inadequate use of antibiotics includes the prescription of inappropriate antibiotics for a brief time, in low or insufficient doses and/or for diseases for which they are not indicated; likewise, excessive therapeutic dosage regimens are also considered inadequate uses of antibiotics [5]. In the specific case of Spain, the consumption of antibiotics for outpatients is above the European average, and in hospitals their prescription is increasing [6].

The inadequate use of antibiotics can be encouraged by self-medication, by the implementation of permissive policies in terms of regulation of the use of these drugs, by the relationship between the healthcare professional and the patient or by the knowledge and attitudes that one has towards them [7,8]. Regarding the latter, while it is true that a poor knowledge or negative attitude towards antibiotics leads inevitably to bad clinical practice, practical skills are not always a reflection of knowledge, a situation known as “theory–practice gap” [9].

To date, the studies which have evaluated the training that healthcare students have about infectious diseases, antimicrobial resistance and safe use of antibiotics have been mainly carried out in students of medicine [7,9] and pharmacy [10,11,12], besides students from other disciplines such as dentistry [13]. However, taking into account that the control of antibiotic resistance requires a multidisciplinary approach, and that nursing staff play a key role in the rational use of antibiotics, as teachers of patients, and as future drug prescribers in our country, the training of nurses could have an important impact in reducing the incidence of antibiotic resistance. Thus, the objective of this paper has been to determine the nursing students’ knowledge and awareness of antibiotic use, resistance and stewardship.

## 2. Results

### 2.1. Description of Sample

A total of 578 students of the Degree of Nursing from the University of Santiago de Compostela (152 of the first year, 133 of the second year, 145 of the third year and 148 of the fourth year) were invited to participate in the study, with a response rate of 59% (66% of the first year, 85% of the second year, 52% of the third year and 36% of the fourth year).

Table 1 shows the demographic characteristics of the participants. Most were women, <25 years old, without antibiotics training prior entering nursing but with family members/close friends working in health-related fields. The students’ favourite practice area to develop their professional activity was, in order of frequency: Emergency, Intensive Care Unit (ICU) and Reanimation Unit (RU); Pediatric nursing and Obstetric-gynecologic nursing.

### 2.2. Knowledge and Awareness Regarding Antibiotic Use, Resistance and Stewardship

The answers to questions about students’ general knowledge (questions 8–19) and specific knowledge and awareness of antibiotics use, resistance and stewardship (questions 24–40) are shown in Table 2. In general, the students had good general knowledge of antibiotics; however, the aspects where there was a greater lack of knowledge were the following: cefotaxime is a cephalosporin (question 10) and antibiotics cannot treat influenza (question 19). On the contrary, the students showed a wide lack of knowledge in terms of antibiotic resistance, despite that 94.5% of the students stated they had heard about antibiotic resistance (question 20), and 45.2% that they had discussed antibiotic resistance topics during the nursing degree (question 21).

The questions with poorer results (<25% of correct answers) were: antibiotic resistance is a natural phenomenon (question 29), beta-lactamase is an enzyme produced by bacteria that can break down aminoglycosides (question 33) and bacteria may acquire efflux pumps that extrude the antibiotic from the cell (question 34).

**Figure 1:**
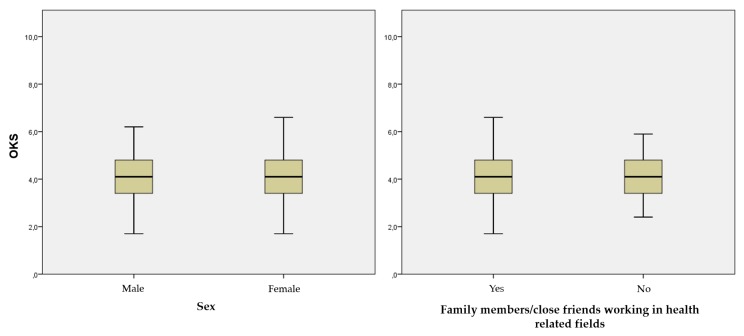
Overall knowledge score (OKS) in accordance with the sex and the existence of family members/close friends working in health-related fields. The overall knowledge score (OKS) was determined according to what has been explained in “Data processing and statistical analysis”. Intergroup differences were analysed using the Mann–Whitney U test. No significant differences were observed (*p* > 0.05).

The overall median knowledge score (general and specific knowledge) was low (4.2 and 95% confidence interval 3.4–4.8), despite the fact that the scores improved during the degree (Table 2). On the contrary, no statistically significant differences were found between male and female students, or between students with or without family members/close friends working in health-related fields (Figure 1).

### 2.3. Perception of Education about Antibiotics

In spite of the 94.5% of students who acknowledged that they had at some point heard about antibiotic stewardship (question 22), only 60.1% remembered having discussed this subject during the training programme in the nursing degree (question 23). Moreover, the students admitted that they had great difficulties in selecting the best antibiotic for a specific infection, knowing how to establish an appropriate regimen of antibiotic therapy or being able to handle patients who demand antibiotic therapy when clearly it is not indicated (Table 3, questions 41, 42, 44). Likewise, the students, who are aware of these limitations, asserted that it would be interesting to have more education on antibiotics and infection control in the nursing degree, especially from the second year onwards (Table 3, questions 46, 47, 50). This idea was reiterated by several students by means of filling out the fifth section of the questionnaire (Table 4).

### 2.4. Psychometric Validation

Validation of the questionnaire involved analysing its reliability (internal consistency and reproducibility) and validity. The Cronbach α value for the knowledge domain was 0.79, indicating acceptable internal consistency. Regarding the test–retest analysis (*n* = 15), the intraclass correlation coefficient for the 15 students who completed the questionnaire twice was excellent, 0.91 (95% confidence interval 0.87–0.93). On the other hand, content validity was demonstrated since the questionnaire was based on expert consensus.

## 3. Discussion

To our knowledge, this is the first study in the literature to analyse the knowledge and awareness of antibiotic use, resistance and stewardship in nursing students. The results have brought to light that while students know general aspects of antibiotics, they have scarce training in antibiotic resistance, an aspect that was confirmed by the students’ deficient perception on the education that they have received about this group of drugs in their degree. However, there should be noted that their knowledge improved with years of training. These results are very useful taking into account that most antibiotic resistance control strategies recommend education for the general population, with nursing staff playing an important role as teachers [14].

An alarming finding of this study is the high percentage of students who are ignorant of the inefficiency of antibiotics to treat viral infections or the typical symptomatology of a common cold, for example: cough, pain. However, this is not an isolated case, since both of these mistakes have been previously notified in different investigations which were carried out among the general population [7,8,15] as well as in healthcare staff [7,11,16]. This lack of knowledge of the indications of antibiotics highlights the need to strengthen this topic in curriculums of students enrolled in health degrees, as it has been referred to by students of medicine [17,18,19,20], pharmacy [10], nursing (current study) and dentistry [13], among other disciplines [13]. It should be important not only when they have to prescribe them, but also to educate the general population.

Antibiotic resistance has important economic consequences and negative repercussions in terms of morbi-mortality. A recent study has revealed that, if we do not take immediate proactive solutions to slow down the rise of antibiotic resistance, in the year 2050 it will be the cause of 10 million deaths and of a loss of 100 trillion USD of economic output [21]. These consequences stress the important need not only to develop new agents to combat multidrug-resistant bacteria [22,23] but also to implement measures to prevent or minimize antibiotic resistance, such as: changing the empiric therapy to the selected therapy in response to the availability of culture and sensitivity results [11], improving hand hygiene practices [18,24], prioritizing the prescription of narrow spectrum antibiotics [25], reducing the use of antibiotics in animals [25], not keeping leftover antibiotics [8], etc. These measures were barely identified correctly by nursing students, which could have important repercussions in clinical practice. We believe that the perception of this deficiency conjoined with the implications that the resistances result in, got students in health degrees, for example medicine [17,18,19,20], pharmacy [10], nursing (current study) and other disciplines [13] to consider that the education they had received during the degree was not enough. 

To date, the studies evaluating knowledge [26,27] and/or awareness [27] of antibiotics in the area of nursing are limited, and the only study which included nursing students was carried out in a Malaysian university [26], so it is very difficult to make comparisons taking into account that the level of training for nurses stipulated in underdeveloped or developing countries is usually inferior to the training stipulated in developed countries [28]. The scarcity of studies about this topic, at least in our country, might be due to the lack of autonomy of nurses when prescribing antibiotics; however, in Spain this situation could change in the near future due to legislative changes that are being introduced. Even so, we cannot forget that the antibiotics administration is a direct responsibility of the nursing staff who have to take into account in clinical practice the biopharmaceutical properties of the antibiotics prescribed, because their effectiveness depends, among other aspects on their concentration and their time of administration [29]. According to students' perceptions, we think that they do not have, or at least they themselves think that they do not have the knowledge to carry out the appropriate use of the antibiotics in clinical practice.

Our study included several limitations. The main limitation of the current study was related to the participation. Despite the fact that it was higher than in other studies [10,18,24,30,31], it was decreasing as students advanced from year to year, which could be associated with the rate of class attendance (higher in the first years of the degree; fourth year students are an exception as they are in practicums all year round). In accordance with this, it would also have been useful to have used an online tool in order to distribute the questionnaire. Second, as students filled in questionnaires themselves, there may be some self-report bias. Third, another limitation of the study would include the absence of information regarding private university students’ knowledge and awareness of antibiotics, as there are not any private universities in Galicia. Because of this limitation, additional studies are needed to determine if the results from our study can be generalized to nursing students with other characteristics (e.g., students who are socioeconomically advantaged).

## 4. Materials and Methods 

### 4.1. Design

An observational cross-sectional descriptive study was carried out. 

### 4.2. Setting and Participants

All the nursing students of the University of Santiago de Compostela (USC, Galicia, Spain), one of the three public universities of Galicia, were invited to participate in the study. Although the students study pharmacology during the second year, we invited all the nursing students in order to use the results from the first course as baseline data and so to check if there was any improvement in knowledge from years of training. The investigation included students enrolled in a nursing course in the academic 2018–2019, of both sexes and 18 years or older who voluntarily accepted to participate. On the contrary, participants who had not studied all the years in the University of Santiago de Compostela were excluded.

The size of the study population was 578 at the time of the research. Keeping the expected frequency of all variables at 50%, the desirable sample size using a 95% confidence interval came out to be 294. However, after 15% inflation and rounding off, the final desired sample size was determined to be 340.

Fifteen days later a small group of 15 students (the same ones who did the pilot study in order to evaluate the clarity and ease of understanding of the questionnaire, see the section “Translation and transcultural adaptation”) repeated the questionnaire for the test–retest reproducibility study.

### 4.3. Translation and Transcultural Adaptation

Translation–backtranslation was the methodology used to make the semantic and cultural adaptation of the questionnaire “knowledge and awareness of antibiotic use, resistance and stewardship (KAAURS)” [10], following the guidelines of Beaton et al. [32] and Sperber et al. [33].

Two translations of the original version of the questionnaire KAAURS were done in Spanish by two bilingual people with wide experience in antimicrobial therapy. In order to verify the adequacy of the translations, these were revised by the investigator team, obtaining a unified version of the questionnaire in Spanish (Supplementary Materials Questionnaire). Then, the backtranslation process was conducted. Following the same procedure, the unified Spanish version was translated again into English by two people, who did not know the original version of the questionnaire. Conceptual and semantic equivalence was analysed for each one of the items. Finally, a pilot study was carried out with 15 students, who did not participate in the final study, in order to evaluate the clarity and ease of understanding of the items. They reported full comprehension of the questions and ease in completing the questionnaire, so no change was carried out.

### 4.4. Data Collection

The information was obtained from the Spanish version (Supplementary Materials Questionnaire) of the questionnaire KAAURS. The questionnaire consists of 50 closed-ended questions (dichotomous and multiple choice), mainly of only one answer, structured into 4 sections. The first section includes demographic data, the second section consists of questions in order to evaluate the general level of knowledge of antibiotics, the third section addresses specific knowledge and awareness regarding antibiotic resistance and stewardship, the fourth section assesses students' perceptions about the education they received on antibiotics, and the fifth section, that is optional allows students to give additional suggestions or opinions about the different topics covered in the study.

The questionnaires were anonymous and self-completed between February and April of 2019. Once the objectives and the purpose of the study were explained, the distribution of the questionnaires was carried out during class break.

### 4.5. Ethical and Legal Considerations

The use of the questionnaire KAAURS was authorized by the author of the original instrument Dr. Inácio. The study was performed with the approval of the Faculty of Nursing, University of Santiago de Compostela. Likewise, after explaining the procedure and the objective of the investigation, we obtained the student’s consent whose participation was completely voluntary. Pursuant to the Declaration of Helsinki and Data Protection Act (Organic Law 3/2018), data confidentiality was guaranteed at all times.

### 4.6. Data Processing and Statistical Analysis

The results were presented as number and percentage. The variable overall knowledge score (OKS) was estimated from the results regarding knowledge (questions 8–19 and 24–40), as it has been described by Inácio et al. [10]. Briefly, the questions regarding knowledge were dichotomized as “correct” and “incorrect”, then the percentage of correct answers for each student was estimated, representing this proportion on a scale between 0 (poor knowledge) and 10 (good knowledge).

Bivariate analysis was performed using Kruskal–Wallis test (for comparisons between the four years of study) and Mann–Whitney U test (for comparisons between genders and students with and without family member/close friend working in health-related fields).

In relation to psychometric properties of the questionnaire, the Cronbach α coefficient was calculated for the knowledge section (questions 8–19 and 24–40) and test–retest reproducibility was studied by intraclass correlation coefficient.

A p-value less than 0.05 was considered significant throughout the study. The software IBM SPSS Statistics (version 24) was used for the statistical processing of the data.

## 5. Conclusions

Future nurses have insufficient knowledge on antibiotics and infection control, so more educational activities are needed within the current nursing curriculum with the aim of achieving a better training level.

## Figures and Tables

**Table 1:** Baseline characteristics of the study’s participants. All the results were expressed as n (%).

Characteristics		Programme					
		First-Year	Second-Year	Third-Year	Fourth-Year	DK/NO	% of the Total
Sex	Male	12 (22.2%)	19 (35.2%)	14 (25.9%)	9 (16.7%)	0 (0%)	54 (15.7%)
	Female	88 (30.4%)	94 (32.5%)	61 (21.1%)	45 (15.6%)	1 (0.3%)	289 (84.3%)
Age	<20	82 (51.9%)	73 (46.2%)	2 (1.3%)	0 (0%)	1 (0.6%)	158 (46.1%)
	20-25	12 (7.3%)	35 (21.2%)	68 (41.2%)	50 (30.3%)	0 (0%)	165 (48.1%)
	>25	6 (31.6%)	5 (26.3%)	5 (26.3%)	3 (15.8%)	0 (0%)	19 (5.5%)
	DK/NO	0 (0%)	0 (0%)	0 (0%)	1 (100%)	0 (0%)	1 (0.3%)
Time living in Spain	<3	2 (28.6%)	2 (28.6%)	3 (42.9%)	0 (0%)	0 (0%)	7 (2%)
	3-6	0 (0%)	0 (0%)	0 (0%)	0 (0%)	0 (0%)	0 (0%)
	>6	97 (29.3%)	108 (32.6%)	71 (21.5%)	54 (16.3%)	1 (0.3%)	331 (96.5%)
	DK/NO	1 (20%)	3 (60%)	1 (20%)	0 (0%)	0 (0%)	5 (1.5%)
Practice area considered	Community nursing	10 (16.9%)	8 (13.6%)	16 (27.1%)	25 (42.4%)	0 (0%)	59 (17.2%)
	Pediatric nursing	39 (27.5%)	43 (30.3%)	42 (29.6%)	18 (12.7%)	0 (0%)	142 (41.4%)
	Obstetric-gynecologic nursing	32 (26.4%)	38 (31.4%)	35 (28.9%)	16 (13.2%)	0 (0%)	121 (35.3%)
	Mental health nursing	17 (29.3%)	15 (25.9%)	15 (25.9%)	11 (19%)	0 (0%)	58 (16.9%)
	Occupational nursing	3 (30%)	2 (20%)	1 (10%)	4 (40%)	0 (0%)	10 (2.9%)
	Geriatric nursing	4 (12.9%)	9 (29%)	8 (25.8%)	10 (32.3%)	0 (0%)	31 (9%)
	Surgical-medical nursing	39 (38.6%)	35 (34.7%)	17 (16.8%)	10 (9.9%)	0 (0%)	101 (29.4%)
	Emergency, ICU and RU	55 (31.8%)	56 (32.4%)	34 (19.7%)	28 (16.2%)	0 (0%)	173 (50.4%)
	Teaching	9 (20.5%)	16 (36.4%)	10 (22.7%)	9 (20.5%)	0 (0%)	44 (12.8%)
	Other	1 (11.1%)	0 (0%)	6 (66.7%)	2 (22.2%)	0 (0%)	9 (2.6%)
	I have not decided yet	22 (28.6%)	28 (36.4%)	16 (20.8%)	10 (13%)	1 (1.3%)	77 (22.4%)
Students with antibiotics training prior entering nursing faculty	Yes	20 (48.8%)	7 (17.1%)	8 (19.5%)	6 (14.6%)	0 (0%)	41 (12%)
	No	80 (26.5%)	106 (35.1%)	67 (22.2%)	48 (15.9%)	1 (0.3%)	302 (88%)
Students with family members/close friends working in health-related fields	Yes	69 (33.8%)	60 (29.4%)	39 (19.1%)	35 (17.2%)	1 (0.5%)	204 (59.5%)
	No	31 (22.5%)	52 (37.7%)	36 (26.1%)	19 (13.8%)	0 (0%)	138 (40.2%)
	DK/NO	0 (0%)	1 (100%)	0 (0%)	0 (0%)	0 (0%)	1 (0.3%)

Abbreviations: DK/NO. Do not know/no opinion; ICU. Intensive Care Unit; RU. Reanimation Unit.

**Table 2:** General and specific knowledge and awareness of antibiotics. All the results were expressed as n (%), except the variable OKS, which was presented as median and confidence interval. The correct answers have been highlighted in bold.

Question (Q)		Programme						
		First-Year	Second-Year	Third-Year	Fourth-Year	DK/NO	% of the Total	*p*
Q8: Amoxicillin is an antibiotic	Disagree	2 (66.7%)	1 (33.3%)	0 (0%)	0 (0%)	0 (0%)	3 (0.9%)	>0.05
	Not sure	39 (81.3%)	7 (14.6%)	2 (4.2%)	0 (0%)	0 (0%)	48 (4%)	
	**Agree**	59 (20.2%)	105 (36%)	73 (25%)	54 (18.5%)	1 (0.3%)	292 (85.1%)	
Q9: Aspirin is an antibiotic	**Disagree**	92 (28.9%)	99 (31.1%)	74 (23.3%)	53 (16.7%)	0 (0%)	318 (92.7%)	>0.05
	Not sure	4 (22.2%)	13 (72.2%)	1 (5.6%)	0 (0%)	0 (0%)	18 (5.2%)	
	Agree	4 (66.7%)	1 (16.7%)	0 (0%)	1 (16.7%)	0 (0%)	6 (1.7%)	
	DK/NO	0 (0%)	0 (0%)	0 (0%)	0 (0%)	1 (100%)	1 (0.3%)	
Q10: Cefotaxime is a cephalosporin	Disagree	1 (25%)	1 (25%)	1 (25%)	1 (25%)	0 (0%)	4 (1.2%)	>0.05
	Not sure	95 (38.3%)	95 (38.3%)	39 (15.7%)	18 (7.3%)	1 (0.4%)	248 (72.3%)	
	**Agree**	4 (4.4%)	17 (18.9%)	35 (38.9%)	34 (37.8%)	0 (0%)	90 (26.2%)	
	DK/NO	0 (0%)	0 (0%)	0 (0%)	1 (100%)	0 (0%)	1 (0.3%)	
Q11:Antibiotics are useful for bacterial infections	Disagree	4 (57.1%)	2 (28.6%)	0 (0%)	0 (0%)	1 (14.3%)	7 (2%)	>0.05
	Not sure	5 (62.5%)	2 (25%)	0 (0%)	1 (12.5%)	0 (0%)	8 (2.3%)	
	**Agree**	91 (27.8%)	108 (33%)	75 (22.9%)	53 (16.2%)	0 (0%)	327 (95.3%)	
	DK/NO	0 (0%)	1 (100%)	0 (0%)	0 (0%)	0 (0%)	1 (0.3%)	
Q12: Antibiotics are useful for viral infections	**Disagree**	78 (27%)	95 (32.9%)	67 (23.2%)	49 (17%)	0 (0%)	289 (84.3%)	>0.05
	Not sure	10 (35.7%)	13 (46.4%)	4 (14.3%)	0 (0%)	1 (3.6%)	28 (8.2%)	
	Agree	12 (46.2%)	5 (19.2%)	4 (15.4%)	5 (19.2%)	0 (0%)	26 (7.6%)	
Q13: Antibiotics are indicated to reduce any kind of pain and inflammation	**Disagree**	64 (24.7%)	81 (31.3%)	68 (26.3%)	46 (17.8%)	0 (0%)	259 (75.5%)	>0.05
	Not sure	13 (32.5%)	17 (42.5%)	6 (15%)	3 (7.5%)	1 (2.5%)	40 (11.7%)	
	Agree	23 (53.5%)	15 (34.9%)	1 (2.3%)	4 (9.3%)	0 (0%)	43 (12.5%)	
	DK/NO	0 (0%)	0 (0%)	0 (0%)	1 (100%)	0 (0%)	1 (0.3%)	
Q14: Antibiotics can cause secondary infections after killing good bacteria present in our organism	Disagree	6 (31.6%)	6 (31.6%)	3 (15.8%)	4 (21.1%)	0 (0%)	19 (5.5%)	>0.05
	Not sure	30 (39%)	21 (27.3%)	16 (20.8%)	10 (13%)	0 (0%)	77 (22.4%)	
	**Agree**	64 (25.9%)	86 (34.8%)	56 (22.7%)	40 (16.2%)	1 (0.4%)	247 (72%)	
Q15: Antibiotics can cause allergic reactions	Disagree	1 (50%)	0 (0%)	0 (0%)	1 (50%)	0 (0%)	2 (0.6%)	>0.05
	Not sure	11 (35.5%)	12 (38.7%)	6 (19.4%)	2 (6.5%)	0 (0%)	31 (9%)	
	**Agree**	88 (28.4%)	101 (32.6%)	69 (22.3%)	51 (16.5)	1 (0.3%)	310 (90.4%)	
Q16: Patients may stop the use of antibiotics as soon as they start feeling better	**Disagree**	88 (28.4%)	105 (33.9%)	66 (21.3%)	50 (16.1%)	1 (0.3%)	310 (90.4%)	>0.05
	Not sure	7 (41.2%)	6 (35.3%)	3 (17.6%)	1 (5.9%)	0 (0%)	17 (5%)	
	Agree	4 (28.6%)	2 (14.3%)	6 (42.9%)	2 (14.3%)	0 (0%)	14 (4.1%)	
	DK/NO	1 (50%)	0 (0%)	0 (0%)	1 (50%)	0 (0%)	2 (0.6%)	
Q17: Colds and coughs should always be treated with antibiotics as patients will recover more quickly	**Disagree**	92 (28.4%)	103 (31.8%)	74 (22.8%)	54 (16.7%)	1 (0.3%)	324 (94.5%)	>0.05
	Not sure	8 (42.1%)	10 (52.6%)	1 (5.3%)	0 (0%)	0 (0%)	19 (5.5%)	
	Agree	0 (0%)	0 (0%)	0 (0%)	0 (0%)	0 (0%)	0 (0%)	
Q18: Antibiotics should always be prescribed as preventive measures to fight against future infections	**Disagree**	92 (30.7%)	89 (29.7%)	67 (22.3%)	51 (17%)	1 (0.3%)	300 (87.5%)	>0.05
	Not sure	6 (21.4%)	19 (67.9%)	2 (7.1%)	1 (3.6%)	0 (0%)	28 (8.2%)	
	Agree	2 (14.3%)	5 (35.7%)	5 (35.7%)	2 (14.3%)	0 (0%)	14 (4.1%)	
	DK/NO	0 (0%)	0 (0%)	1 (100%)	0 (0%)	0 (0%)	1 (0.3%)	
Q19: Antibiotics cannot treat influenza	Disagree	19 (26.4%)	24 (33.3%)	16 (22.2%)	13 (18.1%)	0 (0%)	72 (21%)	>0.05
	Not sure	27 (40.3%)	26 (38.8%)	6 (9%)	8 (11.9%)	0 (0%)	67 (19.5%)	
	**Agree**	54 (26.6%)	63 (31%)	53 (26.1%)	32 (15.8%)	1 (0.5%)	203 (59.2%)	
	DK/NO	0 (0%)	0 (0%)	0 (0%)	1 (100%)	0 (0%)	1 (0.3%)	
Q24: Antibiotic resistance happens when a bacterium loses its sensitivity to an antibiotic	Disagree	6 (40%)	4 (26.7%)	4 (26.7%)	0 (0%)	1 (6.7%)	15 (4.4%)	<0.05 *
	Not sure	16 (44.4%)	8 (22.2%)	8 (22.2%)	4 (11.1%)	0 (0%)	36 (10.5%)	
	**Agree**	77 (26.6%)	101 (34.8%)	63 (21.7%)	49 (16.9%)	0 (0%)	290 (84.5%)	
	DK/NO	1 (50%)	0 (0%)	0 (0%)	1 (50%)	0 (0%)	2 (0.6%)	
Q25: Inappropriate use of antibiotics causes antibiotic resistance	Disagree	2 (66.7%)	0 (0%)	1 (33.3%)	0 (0%)	0 (0%)	3 (0.9%)	<0.05 *
	Not sure	14 (56%)	8 (32%)	2 (8%)	1 (4%)	0 (0%)	25 (7.3%)	
	**Agree**	84 (26.8%)	105 (33.5%)	72 (23%)	51 (16.3%)	1 (0.3%)	313 (91.3%)	
	DK/NO	0 (0%)	0 (0%)	0 (0%)	2 (100%)	0 (0%)	2 (0.6%)	
Q26: Prescribing broad-spectrum antibiotics increases antibiotics resistance	Disagree	2 (11.1%)	6 (33.3%)	6 (33.3%)	4 (22.2%)	0 (0%)	18 (5.2%)	<0.05 *
	Not sure	50 (33.8%)	61 (41.2%)	22 (14.9%)	14 (9.5%)	1 (0.7%)	148 (43.1%)	
	**Agree**	48 (27.3%)	46 (26.1%)	47 (26.7%)	35 (19.9%)	0 (0%)	176 (51.3%)	
	DK/NO	0 (0%)	0 (0%)	0 (0%)	1 (100%)	0 (0%)	1 (0.3%)	
Q27: Poor infection control practices by healthcare professionals cause spread of antibiotic resistance	Disagree	2 (12.5%)	7 (43.8%)	6 (37.5%)	1 (6.3%)	0 (0%)	16 (4.7%)	<0.05 *
	Not sure	43 (47.8%)	30 (33.3%)	12 (13.3%)	4 (4.4%)	1 (1.1%)	90 (26.2%)	
	**Agree**	55 (23.5%)	75 (32.1%)	57 (24.4%)	47 (20.1%)	0 (0%)	234 (68.2%)	
	DK/NO	0 (0%)	1 (33.3%)	0 (0%)	2 (66.7%)	0 (0%)	3 (0.9%)	
Q28: Antibiotics are overused nationally and internationally in healthcare	Disagree	3 (60%)	1 (20%)	1 (20%)	0 (0%)	0 (0%)	5 (1.5%)	>0.05
	Not sure	24 (37.5%)	24 (37.5%)	10 (15.6%)	6 (9.4%)	0 (0%)	64 (18.7%)	
	**Agree**	73 (26.7%)	88 (32.3%)	64 (23.4%)	47 (17.2%)	1 (0.4%)	273 (79.6%)	
	DK/NO	0 (0%)	0 (0%)	0 (0%)	1 (100%)	0 (0%)	1 (0.3%)	
Q29: Appropriate use of antibiotics can cause antibiotic resistance	Disagree	60 (30.5%)	65 (33%)	45 (22.8%)	27 (13.7%)	0 (0%)	197 (57.4%)	<0.05 *
	Not sure	18 (27.3%)	29 (43.9%)	11 (16.7%)	8 (12.1%)	0 (0%)	66 (19.2%)	
	**Agree**	20 (26%)	19 (24.7%)	19 (24.7%)	18 (23.4%)	1 (1.3%)	77 (22.4%)	
	DK/NO	2 (66.7%)	0 (0%)	0 (0%)	1 (33.3%)	0 (0%)	3 (0.9%)	
Q30: Antibiotic stewardship is a phenomenon for which a bacterium gains resistance to an antibiotic	**Disagree**	65 (29.1%)	74 (33.2%)	47 (21.1%)	36 (16.1%)	1 (0.4%)	223 (65%)	>0.05
	Not sure	26 (34.2%)	25 (32.9%)	17 (22.4%)	8 (10.5%)	0 (0%)	76 (22.2%)	
	Agree	9 (20. 9%)	14 (32.6%)	11 (25.6%)	9 (20.9%)	0 (0%)	43 (12.5%)	
	DK/NO	0 (0%)	0 (0%)	0 (0%)	1 (100%)	0 (0%)	1 (0.3%)	
Q31: Exposure to antibiotics appears to be the principal risk factor for appearance of antibiotic-resistant bacteria	Disagree	8 (36.4%)	7 (31.8%)	4 (18.2%)	3 (13.6%)	0 (0%)	22 (6.4%)	>0.05
	Not sure	39 (34.2%)	38 (33.3%)	22 (19.3%)	14 (12.3%)	1 (0.9%)	114 (33.2%)	
	**Agree**	53 (25.9%)	68 (33.2%)	49 (23.9%)	35 (17.1%)	0 (0%)	205 (59.8%)	
	DK/NO	0 (0%)	0 (0%)	0 (0%)	2 (100%)	0 (0%)	2 (0.6%)	
Q32: Antibiotic resistance can be minimized by using narrow-spectrum therapy after identification and susceptibility testing of infectious bacteria	Disagree	2 (25%)	0 (0%)	4 (50%)	2 (25%)	0 (0%)	8 (2.3%)	<0.05 *
	Not sure	60 (39.7%)	51 (33.8%)	21 (13.9%)	18 (11.9%)	1 (0.7%)	151 (44%)	
	**Agree**	37 (20.3%)	62 (34.1%)	50 (27.5%)	33 (18.1%)	0 (0%)	182 (53.1%)	
	DK/NO	1 (50%)	0 (0%)	0 (0%)	1 (50%)	0 (0%)	2 (0.6%)	
Q33: Beta-lactamase is an enzyme produced by bacteria that can break down aminoglycosides	**Disagree**	0 (0%)	1 (100%)	0 (0%)	0 (0%)	0 (0%)	1 (0.3%)	<0.05 *
	Not sure	97 (31.4%)	106 (34.3%)	64 (20.7%)	41 (13.3%)	1 (0.3%)	309 (90.1%)	
	Agree	3 (9.7%)	5 (16.1%)	11 (35.5%)	12 (38.7%)	0 (0%)	31 (9%)	
	DK/NO	0 (0%)	1 (50%)	0 (0%)	1 (50%)	0 (0%)	2 (0.6%)	
Q34. Bacteria may acquire efflux pumps that extrude the antibiotic from the cell	Disagree	2 (33.3%)	2 (33.3%)	1 (16.7%)	1 (16.7%)	0 (0%)	6 (1.7%)	>0.05
	Not sure	86 (29.6%)	99 (34%)	66 (22.7%)	39 (13.4%)	1 (0.3%)	291 (84.8%)	
	**Agree**	12 (27.3%)	11 (25%)	8 (18.2%)	13 (29.5%)	0 (0%)	44 (12.8%)	
	DK/NO	0 (0%)	1 (50%)	0 (0%)	1 (50%)	0 (0%)	2 (0.6%)	
Q35: Use of antibiotics in livestock production and agriculture contributes to antibiotic resistance	Disagree	2 (33.3%)	1 (16.7%)	1 (16.7%)	2 (33.3%)	0 (0%)	6 (1.7%)	>0.05
	Not sure	43 (38.1%)	29 (25.7%)	25 (22.1%)	15 (13.3%)	1 (0.9%)	113 (32.9%)	
	**Agree**	55 (24.9%)	82 (37.1%)	48 (21.7%)	36 (16.3%)	0 (0%)	221 (64.4%)	
	DK/NO	0 (0%)	1 (33.3%)	1 (33.3%)	1 (33.3%)	0 (0%)	3 (0.9%)	
Q36. Improving techniques for bacterial diagnostics will allow combating antibiotic resistance	Disagree	1 (25%)	1 (25%)	1 (25%)	1 (255)	0 (0%)	4 (1.2%)	<0.05 *
	Not sure	17 (47.2%)	12 (33.3%)	4 (11.1%)	3 (8.3%)	0 (0%)	36 (10.5%)	
	**Agree**	82 (27.4%)	99 (33.1%)	70 (23.4%)	48 (16.1%)	0 (0%)	299 (87.2%)	
	DK/NO	0 (0%)	1 (25%)	0 (0%)	2 (50%)	1 (25%)	4 (1.2%)	
Q37: Improved healthcare hygiene helps to control antibiotic resistance	Disagree	11 (27.5%)	18 (45%)	8 (20%)	3 (7.5%)	0 (0%)	40 (11.7%)	<0.05 *
	Not sure	36 (37.1%)	30 (30.9%)	23 (23.7%)	8 (8.2%)	0 (0%)	97 (28.3%)	
	**Agree**	53 (26%)	65 (31.9%)	43 (21.1%)	42 (20.6%)	1 (0.5%)	204 (59.5%)	
	DK/NO	0 (0%)	0 (0%)	1 (50%)	1 (50%)	0 (0%)	2 (0.6%)	
Q38: Today’s research will be sufficient to meet the future needs for new antibiotics	**Disagree**	50 (27.3%)	61 (33.3%)	42 (23%)	30 (16.4%)	0 (0%)	183 (53.4%)	>0.05
	Not sure	34 (33%)	31 (30.1%)	21 (20.4%)	17 (16.5%)	0 (0%)	103 (30%)	
	Agree	16 (28.6%)	21 (37.5%)	12 (21.4%)	6 (10.7%)	1 (1.8%)	56 (16.3%)	
	DK/NO	0 (0%)	0 (0%)	0 (0%)	1 (100%)	0 (0%)	1 (0.3%)	
Q39: Antibiotic resistance will be a greater clinical problem later in my nursing career than it is today	Disagree	1 (20%)	3 (60%)	1 (20%)	0 (0%)	0 (0%)	5 (1.5%)	>0.05
	Not sure	8 (32%)	8 (32%)	8 (32%)	1 (4%)	0 (0%)	25 (7.3%)	
	**Agree**	91 (29.3%)	101 (32.5%)	66 (21.2%)	52 (16.7%)	1 (0.3%)	311 (90.7%)	
	DK/NO	0 (0%)	1 (50%)	0 (0%)	1 (50%)	0 (0%)	2 (0.6%)	
Q40: Formal teaching on proper usage of antibiotics among healthcare students may minimize the phenomena of antibiotic resistance	Disagree	0 (0%)	0 (0%)	0 (0%)	1 (100%)	0 (0%)	1 (0.3%)	>0.05
	Not sure	5 (33.3%)	6 (40%)	0 (0%)	4 (26.7%)	0 (0%)	15 (4.4%)	
	**Agree**	95 (29.3%)	105 (32.4%)	75 (23.1%)	48 (14.8%)	1 (0.3%)	324 (94.5%)	
	DK/NO	0 (0%)	2 (66.7%)	0 (0%)	1 (33.3%)	0 (0%)	3 (0.9%)	
**OKS**		3.8 (3.4-4.5)	4.1 (3.4-4.8)	4.5 (3.8-5.2)	4.8 (4.1-5.2)	3.4 (3.4-3.4)	4.1 (3.4-4.8)	<0.001 *

The overall knowledge score (OKS) was determined according to what has been explained in “Data processing and statistical analysis”. Intergroup differences were analyzed using the Kruskal–Wallis test. * Significant differences (*p* < 0.05). Abbreviations: OKS. Overall knowledge score; DK/NO. Do not know/no opinion.

**Table 3:** Perception about training on antibiotics received in the nursing degree. All the results were expressed as n (%).

Question (Q)		Programme					
		First-Year	Second-Year	Third-Year	Fourth-Year	DK/NO	% of the Total
**Q41:** I have had sufficient pharmacy education to select the best antibiotic for a specific infection	Disagree	87 (29.8%)	93 (31.8%)	65 (22.3%)	46 (15.8%)	1 (0.3%)	292 (85.1%)
	Not sure	11 (27.5%)	14 (35%)	9 (22.5%)	6 (15%)	0 (0%)	40 (11.7%)
	Agree	2 (20%)	5 (50%)	1 (10%)	2 (20%)	0 (0%)	10 (2.9%)
	DK/NO	0 (0%)	1 (100%)	0 (0%)	0 (0%)	0 (0%)	1 (0.3%)
**Q42:** I have had sufficient pharmacy education to select an appropriate regimen (dose, route, frequency) of antibiotic therapy	Disagree	78 (31.2%)	83 (33.2%)	51 (20.4%)	37 (14.8%)	1 (0.4%)	250 (72.9%)
	Not sure	13 (31.7%)	12 (29.3%)	12 (29.3%)	4 (9.8%)	0 (0%)	41 (12%)
	Agree	9 (18%)	17 (34%)	11 (22%)	13 (26%)	0 (0%)	50 (14.6%)
	DK/NO	0 (0%)	1 (50%)	1 (50%)	0 (0%)	0 (0%)	2 (0.6%)
**Q43:** I have had sufficient pharmacy education to understand the mechanisms of antibiotic resistance	Disagree	65 (30%)	72 (33.2%)	44 (20.3%)	35 (16.1%)	1 (0.5%)	217 (63.3%)
	Not sure	25 (37.3%)	20 (29.9%)	17 (25.4%)	5 (7.5%)	0 (0%)	67 (19.5%)
	Agree	10 (17.5%)	19 (33.3%)	14 (24.6%)	14 (24.6%)	0 (0%)	57 (16.6%)
	DK/NO	0 (0%)	2 (100%)	0 (0%)	0 (0%)	0 (0%)	2 (0.6%)
**Q44:** I have had sufficient pharmacy education to handle a patient who demands antibiotics therapy when it is not indicated	Disagree	67 (37%)	61 (33.7%)	33 (18.2%)	19 (19.5%)	1 (0.6%)	181 (52.8%)
	Not sure	17 (23.3%)	19 (26%)	19 (26%)	18 (24.75)	0 (0%)	73 (21.3%)
	Agree	16 (18.2%)	32 (36.4%)	23 (26.1%)	17 (19.3%)	0 (0%)	73 (21.3%)
	DK/NO	0 (0%)	1 (100%)	0 (0%)	0 (0%)	0 (0%)	1 (0.3%)
**Q45:** I have had sufficient pharmacy education regarding appropriate use of antibiotics	Disagree	51 (35.7%)	52 (36.4%)	18 (12.6%)	21 (14.7%)	1 (0.7%)	143 (41.7%)
	Not sure	17 (25%)	27 (39.7%)	13 (19.1%)	11 (16.2%)	0 (0%)	68 (19.8%)
	Agree	32 (25%)	31 (24.2%)	43 (33.6%)	22 (17.2%)	0 (0%)	128 (37.3%)
	DK/NO	0 (0%)	3 (75%)	1 (25%)	0 (0%)	0 (0%)	4 (1.2%)
**Q46:** I would like more education on antibiotics use, resistance and stewardship	Disagree	0 (0%)	2 (100%)	0 (0%)	0 (0%)	0 (0%)	2 (0.6%)
	Not sure	3 (60%)	1 (20%)	0 (0%)	1 (20%)	0 (0%)	5 (1.5%)
	Agree	97 (29%)	108 (32.3%)	75 (22.5%)	53 (15.9%)	1 (0.3%)	334 (97.4%)
	DK/NO	0 (0%)	2 (100%)	0 (0%)	0 (0%)	0 (0%)	2 (0.6%)
**Q47:** I would like more education on microbiology and infection control	Disagree	2 (25%)	4 (50%)	2 (25%)	0 (0%)	0 (0%)	8 (2.3%)
	Not sure	6 (54.5%)	4 (36.4%)	1 (9.1%)	0 (0%)	0 (0%)	11 (3.2%)
	Agree	92 (28.6%)	103 (32%)	72 (22.4%)	54 (15.7%)	1 (0.3%)	322 (93.9%)
	DK/NO	0 (0%)	2 (100%)	0 (0%)	0 (0%)	0 (0%)	2 (0.6%)
**Q48:** Strong knowledge of antibiotics is important in my nursing career	Disagree	0 (0%)	1 (25%)	2 (50%)	1 (25%)	0 (0%)	4 (1.2%)
	Not sure	1 (33.3%)	1 (33.3%)	0 (0%)	1 (33.3%)	0 (0%)	3 (0.9%)
	Agree	99 (29.7%)	108 (32.4%)	73 (21.9%)	52 (15.6%)	1 (0.3%)	333 (97.1%)
	DK/NO	0 (0%)	3 (100%)	0 (0%)	0 (0%)	0 (0%)	3 (0.9%)
**Q49:** Strong knowledge of microbiology and infection control is important in my nursing career	Disagree	1 (33.3%)	1 (33.3%)	0 (0%)	1 (33.3%)	0 (0%)	3 (0.9%)
	Not sure	2 (33.3%)	1 (16.7%)	3 (50%)	0 (0%)	0 (0%)	6 (1.7%)
	Agree	97 (29.3%)	108 (32.6%)	72 (21.8%)	53 (16%)	1 (0.3%)	331 (96.5%)
	DK/NO	0 (0%)	3 (100%)	0 (0%)	0 (0%)	0 (0%)	3 (0.9%)
**Q50:** When do you think your nursing school should spend more time teaching about antibiotics? (Please select all that apply)	First- year	30 (46.2%)	23 (35.4%)	5 (7.7%)	7 (10.8%)	0 (0%)	65 (19%)
	Second-year	73 (30.2%)	89 (36.8%)	47 (19.4%)	32 (13.2%)	1 (0.4%)	242 (70.6%)
	Third-year	52 (26%)	61 (30.5%)	47 (23.55)	40 (20%)	0 (0%)	200 (58.3%)
	Fourth-year	17 (26.2%)	20 (30.8%)	14 (21.5%)	14 (21.5%)	0 (0%)	65 (19%)
	Never	1 (33.3%)	1 (33.3%)	1 (33.3%)	0 (0%)	0 (0%)	3 (0.9%)

Abbreviations: DK/NO. Don’t know/no opinion.

**Table 4:** Suggestions and opinions given by nursing students concerning the topics presented in the questionnaire.

Commentary	n (% of the Total)
The subject of antibiotics is very important, more training is needed	12 (3.5%)
More knowledge on microbiology and infectious diseases is needed	4 (1.2%)
The organization of lectures, courses and/or awareness campaigns on this subject would be interesting	2 (0.6%)
I still have not received any information so as to answer some questions	4 (1.2%)
It would be necessary to have knowledge about the different drugs before carrying out the practicums.	1 (0.3%)
Others	8 (2.3%)
Total	32 (9.4%)

## Supplementary Materials

The following are available online at https://www.mdpi.com/2079-6382/8/4/203/s1, Questionnaire: Cuestionario sobre conocimiento y conciencia de los estudiantes de Grado de Enfermería sobre el uso, resistencia y administración de antibióticos.
