# Supplementary material for: Nursing Students’ Knowledge and Awareness of Antibiotic Use, Resistance and Stewardship: A Descriptive Cross-Sectional Study
Source: Antibiotics (Basel). 2019 Oct 30;8(4):203. doi: 10.3390/antibiotics8040203 (PMC6963445; doi:10.3390/antibiotics8040203)
Supplement: Supplementary file 1 [file antibiotics-08-00203-s001.pdf]

Appendix A. Cuestionario sobre conocimiento y conciencia de los estudiantes de Grado de Enfermería sobre el uso, resistencia y administración de antibióticos.

PRIMERTA PARTE: DATOS DEMOGRÁFICOS

1. Sexo: □ Masculino □ Femenino

2. Edad (en años): □ <20 □ 20-25 □ >25

3. ¿Cuánto tiempo lleva viviendo en España (en años)?: □ <3 □ 3-6 □ >6

4. ¿Qué año del Grado de Enfermería está usted cursando actualmente?:
□ 1º □ 2º □ 3º □ 4º

5. ¿En qué área de enfermería está considerando trabajar cuando se gradúe? (Por favor seleccione todas las que considere):

Enfermería comunitaria.
Enfermería pediátrica.
Enfermería obstétrico-ginecológica.
Enfermería de la salud mental.
Enfermería del trabajo.
Enfermería geriátrica.
Enfermería médico-quirúrgica.
Urgencias, UCI, REA.
Enseñanza.
Otras áreas de enfermería. Indique cual:
…………………………….
No lo he decidido todavía.

6. ¿Ha hecho alguna investigación o ha recibido algún tipo de formación sobre antibióticos antes de entrar en el Grado de Enfermería?: □ Sí □ No

7. ¿Tiene usted algún familiar o amigo cercano que trabaje en un campo relacionado con las ciencias de la salud?: □ Sí □ No

SEGUNDA PARTE: CONOCIMIENTOS GENERALES SOBRE ANTIBIÓTICOS

Por favor, díganos si está de acuerdo o en desacuerdo con cada uno de los siguientes enunciados:

8. La amoxicilina es un antibiótico.
□ En desacuerdo □ No estoy seguro □ De acuerdo

9. La aspirina es un antibiótico.
□ En desacuerdo □ No estoy seguro □ De acuerdo

10. La cefotaxima es una cefalosporina.
□ En desacuerdo □ No estoy seguro □ De acuerdo

11. Los antibióticos están indicados en el tratamiento de infecciones bacterianas.
□ En desacuerdo □ No estoy seguro □ De acuerdo

12. Los antibióticos están indicados en el tratamiento de infecciones víricas.
□ En desacuerdo □ No estoy seguro □ De acuerdo

13. Los antibióticos están indicados para el tratamiento del dolor o inflamación.
□ En desacuerdo □ No estoy seguro □ De acuerdo

14. Los antibióticos pueden causar infecciones secundarias por matar bacterias beneficiosas presentes en nuestro organismo.
□ En desacuerdo □ No estoy seguro □ De acuerdo

15. Los antibióticos pueden provocar reacciones alérgicas.
□ En desacuerdo □ No estoy seguro □ De acuerdo

16. Los pacientes pueden suspender el tratamiento antibiótico a medida que se vayan encontrando mejor.
□ En desacuerdo □ No estoy seguro □ De acuerdo

17. Los resfriados y la tos siempre deben ser tratados con antibióticos, pues los pacientes se recuperan más rápidamente.
□ En desacuerdo □ No estoy seguro □ De acuerdo

18. Los antibióticos deberían ser prescritos siempre como medida preventiva para combatir futuras infecciones.

□ En desacuerdo □ No estoy seguro □ De acuerdo

19. Los antibióticos no se pueden usar para el tratamiento de la gripe.

□ En desacuerdo □ No estoy seguro □ De acuerdo

TERCERA PARTE: CONOCIMIENTO Y CONCIENCIA SOBRE LA RESISTENCIA Y ADMINISTRACIÓN DE ANTIBIÓTICOS

20. ¿Ha oído hablar alguna vez de la resistencia antibiótica?: □ Sí □ No

21. Concretamente ¿Se ha tratado alguna vez el tema de la resistencia antibiótica en el Grado de Enfermería?: □ Sí □ No

22. ¿Ha oído hablar alguna vez de la administración de antibióticos?: □ Sí □ No

23. Concretamente, ¿Se ha tratado alguna vez el tema de la administración antibiótica durante el Grado de Enfermería?: □ Sí □ No

Por favor, díganos si está de acuerdo o en desacuerdo con cada uno de los siguientes enunciados:

24. La resistencia antibiótica aparece cuando una bacteria pierde su sensibilidad frente a un antibiótico.

□ En desacuerdo □ No estoy seguro □ De acuerdo

25. El uso inapropiado de los antibióticos produce resistencia antibiótica.

□ En desacuerdo □ No estoy seguro □ De acuerdo

26. Prescribir antibióticos de amplio espectro aumenta el riesgo de desarrollar resistencia antibiótica.

□ En desacuerdo □ No estoy seguro □ De acuerdo

27. Prácticas deficientes en el control de la infección por parte de profesionales de la salud contribuyen a la propagación de la resistencia antibiótica.

□ En desacuerdo □ No estoy seguro □ De acuerdo

28. Existe una prescripción excesiva de antibióticos a nivel nacional e internacional.

□ En desacuerdo □ No estoy seguro □ De acuerdo

29. El uso apropiado de antibióticos puede causar resistencia antibiótica.

□ En desacuerdo □ No estoy seguro □ De acuerdo

30. La administración de antibióticos es el proceso por el cual una bacteria adquiere resistencia a un antibiótico.

□ En desacuerdo □ No estoy seguro □ De acuerdo

31. La exposición a antibióticos parece ser el principal factor de riesgo para la aparición de bacterias resistentes a los mismos.

□ En desacuerdo □ No estoy seguro □ De acuerdo

32. La resistencia antibiótica puede ser minimizada utilizando terapias de espectro reducido tras la identificación y evaluación de la susceptibilidad de la bacteria infecciosa.

□ En desacuerdo □ No estoy seguro □ De acuerdo

33. La beta-lactamasa es una enzima producida por bacterias que puede romper aminoglucósidos.

□ En desacuerdo □ No estoy seguro □ De acuerdo

34. Las bacterias pueden desarrollar bombas de eflujo para expulsar al antibiótico de la célula.

□ En desacuerdo □ No estoy seguro □ De acuerdo

35. El uso de antibióticos en la producción de ganado y agricultura contribuye a la resistencia antibiótica.

□ En desacuerdo □ No estoy seguro □ De acuerdo

36. Mejorar las técnicas de identificación bacteriana permitirá combatir la resistencia antibiótica.

□ En desacuerdo □ No estoy seguro □ De acuerdo

37. La mejora en la higiene sanitaria ayuda a controlar la resistencia antibiótica.

□ En desacuerdo □ No estoy seguro □ De acuerdo

38. Las investigaciones actuales serán suficientes para conocer las futuras necesidades de nuevos antibióticos.

□ En desacuerdo □ No estoy seguro □ De acuerdo

39. La resistencia antibiótica será un problema clínico mayor con el paso de los años.

□ En desacuerdo □ No estoy seguro □ De acuerdo

40. La enseñanza oficial de la debida utilización de antibióticos entre estudiantes sanitarios puede minimizar el fenómeno de la resistencia antibiótica.

□ En desacuerdo □ No estoy seguro □ De acuerdo

CUARTA PARTE: PERCEPCIÓN DE LA EDUCACIÓN SOBRE ANTIBIÓTICOS RECIBIDA EN EL GRADO DE ENFERMERÍA

Por favor, díganos si está de acuerdo o en desacuerdo con cada uno de los siguientes enunciados:
He recibido suficiente formación para:

- 41. Saber seleccionar el mejor antibiótico frente a una determinada infección.

  □ En desacuerdo □ No estoy seguro □ De acuerdo
- 42. Establecer un régimen adecuado (dosis, rutina, frecuencia) de terapia antibiótica.

  □ En desacuerdo □ No estoy seguro □ De acuerdo
- 43. Comprender los mecanismos de resistencia antibiótica.

  □ En desacuerdo □ No estoy seguro □ De acuerdo
- 44. Manejar un paciente que demanda terapia antibiótica cuando realmente no está indicada.

  □ En desacuerdo □ No estoy seguro □ De acuerdo
- 45. Comprender la utilización adecuada de los antibióticos.

  □ En desacuerdo □ No estoy seguro □ De acuerdo

Me gustaría tener más formación sobre:

- 46. El uso de antibióticos, resistencias y administración.

  □ En desacuerdo □ No estoy seguro □ De acuerdo
- 47. Microbiología y control de la infección.

  □ En desacuerdo □ No estoy seguro □ De acuerdo

Un amplio conocimiento de los siguientes temas es importante en mi carrera como enfermera:

- 48. Antibióticos.

  □ En desacuerdo □ No estoy seguro □ De acuerdo
- 49. Microbiología y control de las infecciones.

  □ En desacuerdo □ No estoy seguro □ De acuerdo

50. ¿En qué curso cree que el Grado de Enfermería debería invertir más tiempo en formar sobre antibióticos? (Seleccione todas las que considere):

□ 1º □ 2º □ 3º □ 4º □ Ninguno de los anteriores

QUINTA PARTE: COMENTARIO FINAL

Por favor, déjenos aquí cualquier información adicional, sugerencias u opiniones que quiera compartir con nosotros acerca de los temas presentados en el cuestionario (opcional):
